# O065. Drug-resistant chronic cluster headache successfully treated with supraorbital plus occipital nerve stimulation. A rare case report

**DOI:** 10.1186/1129-2377-16-S1-A97

**Published:** 2015-09-28

**Authors:** Marco Mercieri, Andrea Negro, Barbara Silvestri, Lidia D'Alonzo, Sara Tigano, Roberto Arcioni, Paolo Martelletti

**Affiliations:** Department of Medical-Surgical Sciences and Traslational Medicine, Sapienza University of Rome, Sant'Andrea Hospital, Rome, Italy; Department of Clinical and Molecular Medicine, Sapienza University of Rome, Sant'Andrea Hospital, Rome, Italy

Chronic cluster headache (CCH) is a rare and extremely disabling headache syndrome with a recent clinical systematization of its clinical frame from the European Headache Federation[1]. We present a case of a young man affected by drug-resistant chronic CH (rCCH) who showed improvement after a two-time combined supraorbital and occipital nerve stimulation (S-ONS). The clinical improvement was still present at 6-month follow-up.

A 37-year-old man (LB), with an 18-year history of episodic CH *ab initio,* was referred to our Regional Referral Headache Centre first, and then to the Pain Unit, because the headache had become chronic (CCH), and the patient suffered from daily cluster attacks (4-6/day). The patient became progressively unresponsive to prophylactic/acute therapies, including O_2_-therapy, verapamil up to 600 mg/day, external vagus nerve stimulation, etc.

The headache was strictly left-sided. The pain emerged at the left occipital region and migrated towards the ipsilateral eye, becoming throbbing and increasing up to 10/10. It remained fixed within the orbital area between 45 and 80 minutes, accompanied by concurrent, ipsilateral conjunctival injection and tearing. Brain magnetic resonance imaging excluded any underlying lesions.

Sumatriptan 6 mg i.m. maintained over time its efficacy, but during the last year the risk of serious cardiac side effects suggested to look for an alternative treatment to reduce its harmful overuse.

After careful psychological assessment, the patient was considered eligible for occipital nerve stimulation (ONS) trial. Three weeks later he was implanted with a >50%/<85% reduction of attack number and intensity of pain. Unfortunately, three months after surgery, the patient complained of a reappearance of his usual severe CH attack (VAS 10), periorbitally located. We decided to implant an additional electrocatheter stimulating bilaterally the supraorbital nerves (SON). At six-month follow-up the patient referred suffering of 1 attack a week, of mild/moderate intensity, not altering his overall improved quality of life.

ONS was efficient in most of the rCCH patients reported in the literature with low complication rates[2]. In our patient, the ONS was partially effective in relieving symptoms, achieving excellent pain relief only when supraorbital stimulation (SON) was associated [Fig. 1]. Evidence from future RCTs should support this approach in order to give guidelines for a multimodal approach to similar rare/unusual case reports.

**Figure 1:**
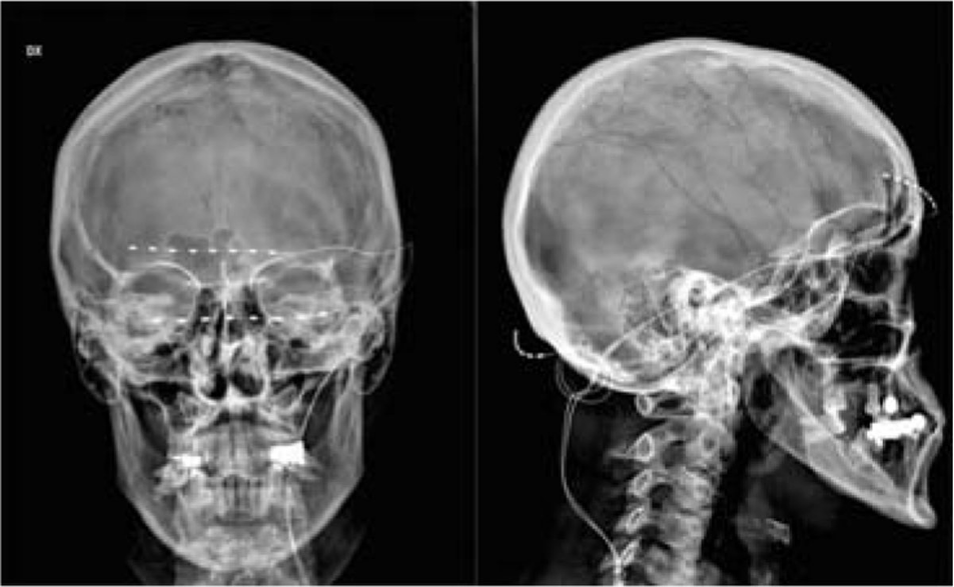
Radiographic image revealing multiple - ONS plus SNO - implant in rCCH patient.

Written informed consent to publication was obtained from the patient(s).

## Conflicts of interests

The authors state that there were no conflicts of interests in respect to the work reported in the paper. IPG and the electrocatheters were paid by the National Heath System as compassionate therapy.
